# Large-Scale Examination of Spatio-Temporal Patterns of Drifting Fish Aggregating Devices (dFADs) from Tropical Tuna Fisheries of the Indian and Atlantic Oceans

**DOI:** 10.1371/journal.pone.0128023

**Published:** 2015-05-26

**Authors:** Alexandra Maufroy, Emmanuel Chassot, Rocío Joo, David Michael Kaplan

**Affiliations:** 1 Institut de Recherche pour le Développement, UMR MARBEC (CNRS/IRD/Ifremer/UM2), Avenue Jean Monnet, Sète Cédex France; 2 Institut de Recherche pour le Développement, UMR MARBEC (CNRS/IRD/Ifremer/UM2), SFA, Fishing Port, Victoria, Seychelles; 3 IMARPE, Esquina Gamarra y General Valle S/N Chucuito, Callao, Lima, Peru; 4 Virginia Institute of Marine Science, College William and Mary, Gloucester Point, Virginia, United States; Deakin University, AUSTRALIA

## Abstract

Since the 1990s, massive use of drifting Fish Aggregating Devices (dFADs) to aggregate tropical tunas has strongly modified global purse-seine fisheries. For the first time, a large data set of GPS positions from buoys deployed by French purse-seiners to monitor dFADs is analysed to provide information on spatio-temporal patterns of dFAD use in the Atlantic and Indian Oceans during 2007-2011. First, we select among four classification methods the model that best separates “at sea” from “on board” buoy positions. A random forest model had the best performance, both in terms of the rate of false “at sea” predictions and the amount of over-segmentation of “at sea” trajectories (i.e., artificial division of trajectories into multiple, shorter pieces due to misclassification). Performance is improved via post-processing removing unrealistically short “at sea” trajectories. Results derived from the selected model enable us to identify the main areas and seasons of dFAD deployment and the spatial extent of their drift. We find that dFADs drift at sea on average for 39.5 days, with time at sea being shorter and distance travelled longer in the Indian than in the Atlantic Ocean. 9.9% of all trajectories end with a beaching event, suggesting that 1,500-2,000 may be lost onshore each year, potentially impacting sensitive habitat areas, such as the coral reefs of the Maldives, the Chagos Archipelago, and the Seychelles.

## Introduction

It has been known for millennia that objects drifting at the surface of the ocean, hereafter referred to as drifting Fish Aggregating Devices (dFADs), attract various species of fish, though the reasons for this behaviour remain poorly understood [1,2]. Fishers have used dFADs for centuries as indicators of higher abundance, better catchability, increased fish school size and ultimately to facilitate the capture of fish [1,3]. Originally, dFADs were either natural marine objects, such as algae or marine mammals, or terrestrial wooden debris, e.g. entering the ocean through river mouths [4]. Since the late 1980s, however, the use of man-made dFADs by pelagic purse-seine fleets has become widespread. They generally consist of a bamboo raft covered with old pieces of purse seine netting and vertical filaments also made of netting hanging down beneath the raft serving as a subsurface drogue (up to 100 m) [5–8]. In tropical tuna fisheries, artificial dFADs have become increasingly important over time and annual global purse seine tuna catches on dFADs reached more than 1.5 million tons in the last decade [9,10]. The massive development of the dFAD-associated fishery has introduced major changes to the efficiency and selectivity of purse seiners that are not well reflected in traditional indices of fishing effort, such as time-at-sea or search-time. This has hindered the use of purse-seine catch rates for the estimation of tuna abundances needed for stock assessment [10,11]. In addition, the extensive use of dFADs has raised serious concerns regarding increased bycatch and juvenile catch, reductions in tuna survival and fitness, and changes in ecosystem functioning [10,12–14]. Despite these concerns, little information has previously been available on dFAD use worldwide. Such information is crucial to monitoring and management of the impacts of dFADs on pelagic ecosystems. As a result, Tuna Regional Fisheries Management Organisations (T-RFMOs) have recently called for dFAD management plans, including data collection on deployment and use of dFADs by purse seiners and supply vessels (e.g. Resolution 12/08 of the IOTC, [15]).

Here, we present the first detailed, spatially-extensive treatment and analysis of the use of dFADs by purse-seiners in the Indian and Atlantic Oceans. We focus on the French component of the fishery, representing an annual catch of about 125,000 t, more than 20% of the total catch on dFADs in these oceans [16,17]. French tuna purse seiners began to build and deploy artificial drifting bamboo rafts equipped with radio-range transmitters in the late 1980s [18]. Detailed records of the positions of floating objects only became available with the emergence of GPS-equipped, satellite-linked buoys in the late 1990s which were coupled to a GIS software system onboard the vessels to monitor dFAD positions in near real-time. However, despite the intensification of dFAD fishing, information on buoy positions has remained highly confidential until recently. Under an agreement with the French purse seine fleet, we have obtained detailed dFAD tracking information for the period 2007–2011 from the 3 French purse seine fishing companies operating in the Atlantic and Indian Oceans.

Our objectives here are (1) to develop the baseline methodology for treatment and analysis of dFAD GPS positions and (2) to carry out an initial examination of dFAD spatio-temporal use and potential impacts. As dFADs data contain both positions while the dFAD was onboard the purse-seine vessel and positions while the dFAD was drifting at sea, four discriminative classification methods are compared for their ability to correctly identify dFAD drift phases on a subset of the data with known state. The classification method with the highest performance is then applied to the full dataset and used to describe the spatio-temporal patterns of dFAD use by a major component of the tropical tuna purse seine fishery in the Atlantic and Indian Oceans. Classified data serve as a basis (i) to identify dFAD density hotspots and measure time and distance at sea, all essential to understanding the impacts of an array of floating objects on tuna stocks and pelagic ecosystems, (ii) to detect dFAD beaching events and their corresponding deployment positions so as to evaluate potential damage to fragile coastal ecosystems and propose management strategies, and (iii) to identify “ineffective” or “ghost” dFAD fishing effort as characterized by dFADs moving out of established fishing areas.

## Material and Methods

### Fisheries data and FAD use overview

Data on catch and effort of French purse seiners have been collected by the ‘Institut de Recherche pour le Développement’ (IRD) since the development of the fishery in the Atlantic and Indian oceans in the early 1970s and 1980s, respectively. For the present study, fine-scale operational data based on skipper logbooks were available for the period 2007–2011. They describe the activities of purse seiners, the association type of tuna schools detected (i.e. free swimming or dFAD-associated school), the positions of purse seine fishing sets, as well as the tonnage and commercial size categories of tuna catches. Similar catch and effort data are available for other components of the purse-seine fishery, notably the Spanish fleet, on a 1° lon-lat grid. In addition, French purse seiners have been equipped with Vessel Monitoring Systems (VMS) since the early 2000s as part of the monitoring, control, and surveillance program of the European Union. The GPS position of each vessel is recorded on an hourly basis, enabling construction of vessel-specific trajectories over their typical 4–6 week fishing trips. This data can be used as a complement to buoy position data, in particular to help identify time periods when buoys were not in the ocean.

Before discussing dFAD position data, it is important to understand how dFADs are used by fishers. When leaving the port, purse seiners bring on board GPS buoys, bamboo rafts and/or the necessary material to build them. These will be used to maintain an array of dFADs belonging to the vessel. This can be done either by deploying new dFADs equipped with GPS buoys, equipping natural floating objects with GPS buoys or appropriating a floating object owned by another vessel by replacing its GPS buoy. Activities related to dFADs and buoys can also be conducted by auxiliary vessels that generally collaborate with 1–2 purse seiners [19]. GPS buoys are turned on before being deployed on a floating object to assure they are functioning correctly. During this period, which can last from a few hours to a few weeks, the GPS signal, transmitted via satellite through systems such as Inmarsat D+ or Iridium, is a sequence of “on board” positions that are similar to VMS positions of the fishing vessel. GPS buoys are then deployed on dFADs for a period of days to months during which time tuna may aggregate under the dFAD. When the level of aggregation is acceptable, a fishing set may be undertaken, either by the deploying vessel, or any vessel that has detected the tuna school. During the fishing set, the dFAD and/or GPS buoy may be retrieved or left at sea. GPS buoy tracks are therefore a succession of “on board” and “at sea” positions. Whereas GPS buoys belong to a single vessel, they may be moved from one floating object to another several times over their life-cycle, be retrieved and changed by foreign vessels operating in the same zone, and the objects they are attached to may be used by multiple vessels.

All these activities occur on fishing grounds that are common to European (mainly France and Spain) and Asian purse seine fleets, either in the open ocean or in Economic Exclusive Zones (EEZ) through fishing licenses. In the Indian Ocean, purse-seine fishing is highly seasonal with a primary peak of activity on floating objects during the third quarter of the year when the fleet concentrates off the coast of Somalia and a secondary peak from March to May when the fleets concentrates in the Mozambique Channel [20]. In the Atlantic Ocean, the seasonality in dFAD activities is less important but a low season occurs from June to August [6].

Once deployed, dFADs share many characteristics with typical Lagrangian drifters used in oceanographic studies, but differ in several important ways. First, the drogue beneath the dFAD is longer than what is typically used in oceanographic studies (up to 100 m). This generally slows the movement of dFADs with respect to surface currents, which is considered desirable by fishers for successful aggregation of tunas. Second, the technology of dFAD tracking buoys is somewhat different, including the use of electronic protection keys to prevent use of the buoys by non-owner vessels, solar panels to increase the buoy battery lifetime, and two-way satellite communications so that the frequency of emission of the GPS signal can be remotely controlled by the owning vessel [2,9,21].

### dFAD GPS buoy data and pre-processing

**Fig 1:**
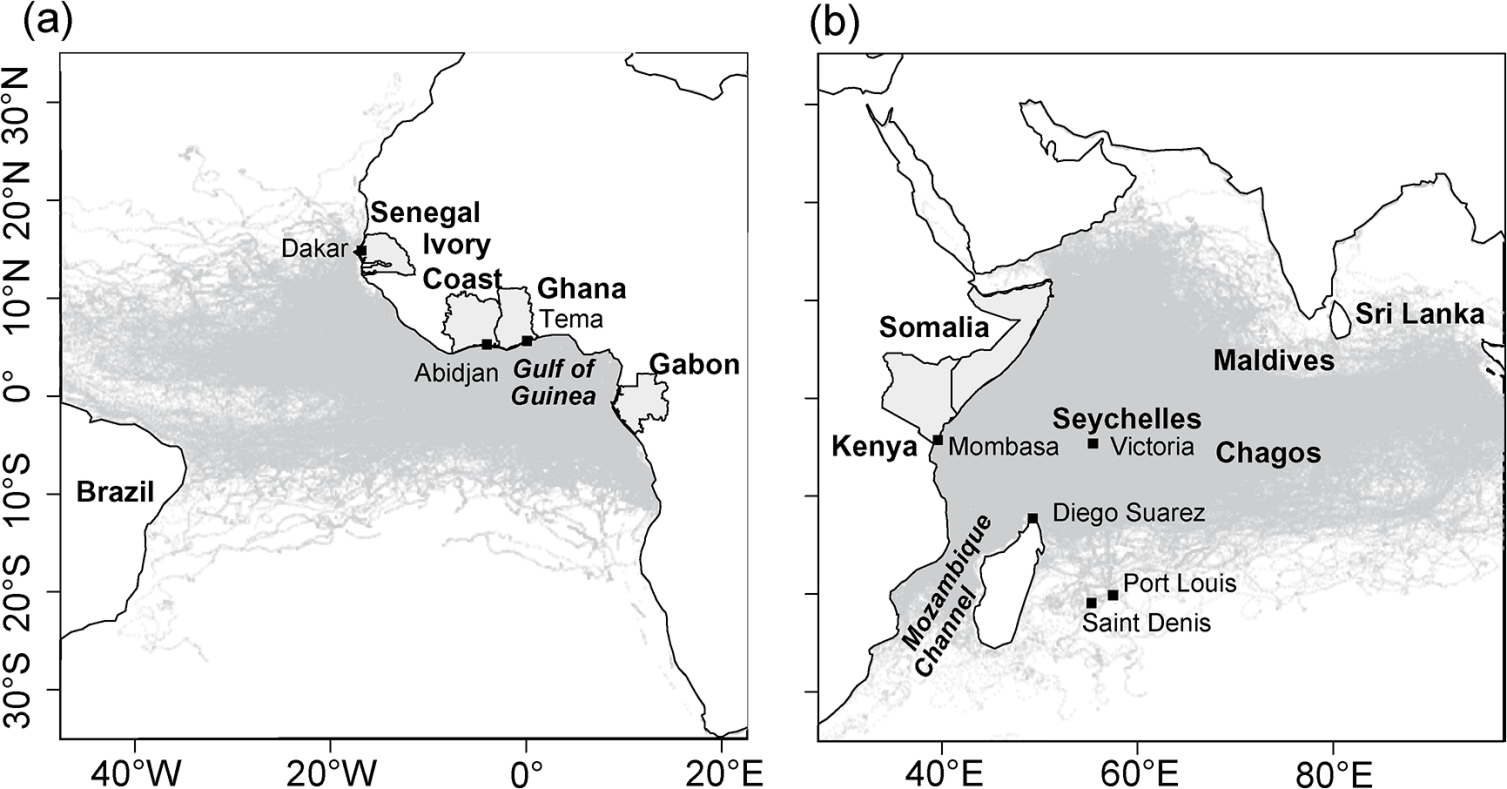
Location of raw GPS buoy positions in the Atlantic (a) and Indian (b) Oceans from January 2007 to December 2011.

dFAD GPS buoy raw data provided by the 3 French fishing companies consist of GPS positions described by latitude and longitude, time of acquisition of the GPS signal (date and hour, with no information on the minute of acquisition of the signal), a vessel identifier and surface water temperature (°C). Timesteps between consecutive data points are irregular (i.e., 1 h, 6 h, 12 h, 1 d or more) depending on the intended use of the dFAD at a given time (e.g.when a fishing vessel intends to visit a given dFAD, it reduces the time between two emissions of a GPS buoy to 1 hour). Several vessels can monitor the same buoy during the same hour, resulting in repeated space-time positions. Furthermore, because time was only recorded to hours (i.e., minutes and seconds were not recorded) in the raw data, a similar time of emission can refer to several different positions during the same hour. We eliminated duplicate timesteps by calculating a unique position as the geographic midpoint of the different positions available for a given hour. Rare records without a valid latitude or longitude were eliminated. Buoys also occasionally erroneously produce two consecutive identical positions separated by a finite period of time. These “doubled” positions can produce inconsistent, extremely-high perceived speeds (reaching sometimes 100 m.s^-1^) between the second repeated position and the position immediately after it. We eliminated such repeated positions, keeping only the first of the two identical positions. The resulting dataset is stored in a PostGreSQL 9.1.9/PostGIS 2.0.1 database, and includes approximately 1,741,000 positions from 9,289 buoys used by 29 purse seiners operating in the Atlantic and Indian Oceans during the period 2007–2011 (Fig 1). The fraction of purse seiners and auxiliary vessels that have provided GPS buoy positions varies between years and fishing companies, with a gradual increase towards 100% coverage of French fishing vessels in recent years (Table 1).

**Table 1:** Yearly proportion of vessels of the French purse seine fishing fleet for which information on GPS buoys was available during 2007–2011 in the Atlantic Ocean (AO) and Indian Ocean (IO).

**Year**	**AO**	**IO**	**Coverage (%)**
2007	3/5	16/19	79.2%
2008	5/7	16/19	87.5%
2009	7/10	14/18	75%
2010	10/10	13/13	100%
2011	9/9	13/13	100%

Note that 100% coverage means that 100% of the fishing vessels have provided data but not that they have provided data for the totality of their GPS buoys.

Given the complex utilisation of dFADs and GPS buoys described in the previous section, it is useful to define specific terminology for different parts of the dFADs positions dataset. Position data are referred to as “GPS buoy positions”. The term “GPS buoy track” is used to refer to the ensemble of positions belonging to a single GPS buoy. Tracks are broken down into “on board” and “at sea” trajectories, consisting of sequences of positions classified as having a consistent state. “At sea” trajectories are also referred to as “dFAD trajectories” or “dFAD positions” as these correspond to periods the GPS buoy is generally attached to a dFAD.

### Construction of the learning dataset

**Fig 2:**
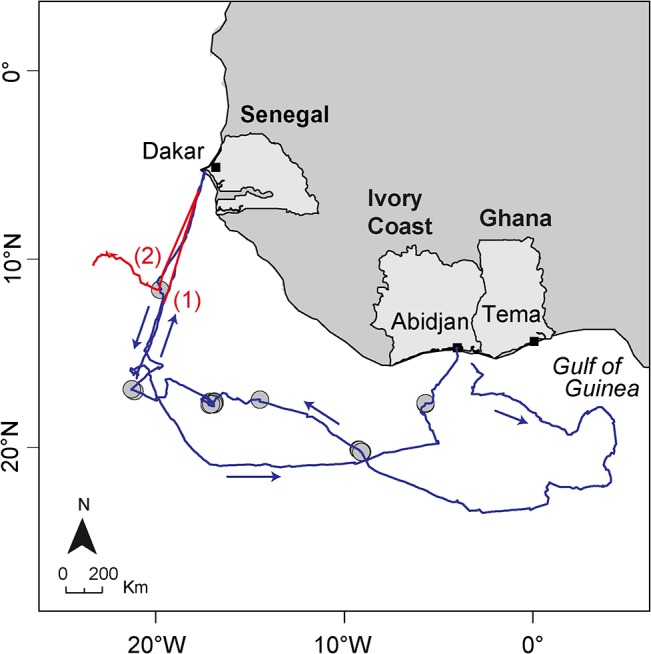
Example of vessel (blue line) and buoy (red line) trajectories inferred from VMS and buoy GPS positions, respectively. After leaving the port of Abidjan (black square) the boat heads to the East in the direction of the Gulf of Guinea, before heading to the West in the direction of Dakar and conducting a series of fishing sets (grey dots). The overlap of the buoy and vessel trajectories indicates that the vessel turned on this particular buoy (1) before entering the port of Dakar. The buoy was likely deployed after leaving the port, shortly after performing a fishing set (2).

The true state of a subset of the GPS positions available was manually determined using complementary fishery data. Vessels trajectories were inferred from VMS position measurements and superimposed in space and time on GPS buoy trajectories to detect shared pieces of tracks (Fig 2). VMS tracks possessing positions close in space (<5 km) and time (<1 d) to GPS buoy positions were initially selected for closer comparison. These “nearby” VMS trajectories were interpolated at the emission times of GPS buoys, and distances between buoys and fishing vessels at identical times were calculated. Original buoy tracks and nearby VMS tracks, spatial separations between the two, buoy speeds, and locations of fishing sets were simultaneously visualized using Matlab [22]. Points of bifurcation between GPS buoy and VMS tracks, as well as the nature of the GPS buoy track preceding and following these bifurcations (e.g., consistently low or high speeds, and sinuous versus straightline tracks), were used to assign “on board” (B) or “at sea” (S) states to individual buoy positions. Geographic locations of the principal tuna landing ports were used to classify positions less than 5 km from a port as “on board” positions. Buoy positions too distant in time (>1 d) and space (>5 km) from any VMS or fishing set data and having speeds that were consistently too large (> 1.5 m.s^-1^) to be considered “at sea” (possible if the buoy was recuperated by a non-French purse-seiner for which we do not have VMS data) were not assigned a class. Variables such as buoy speed or distance to the nearest port, that were used later to build the classification models, were only used as a complementary source of information. For example, it was sometimes difficult to visually determine a transition from “on board” to “at sea”. In such cases, if buoy speed decreased between time t and t+1, then position at time t was assigned a class “on board” and “at sea” at t+1. A total of 19,927 points corresponding to 207 different buoy trajectories were classified using this method (2.3% of the buoy dataset). The majority of this learning dataset consisted of “at sea” positions, with 13.8% of the learning dataset classified as “on board” positions.

### Classification model selection

Four binary classification methods were compared for their ability to correctly predict the “at sea” (S) or “on board” (B) state of each dFAD position based on a set of predictor variables characterizing buoy speed, acceleration, time step, water temperature, etc. at each position (Table 2). The intended, long-term use of classification models is to optimally classify new dFAD position data received from the fishing companies on a quarterly basis. As the resulting large dFAD dataset will be used by multiple individuals having disparate levels of statistical expertise, it is desirable to identify the simplest, most-computationally-efficient classification method that can accurately predict buoy state. Therefore, although one would expect that more sophisticated classification methods (e.g., random forests) will perform best, simpler methods were also tested to assess trade-offs in terms of accuracy and computational time associated with different levels of model complexity.

**Table 2:** List of predictor variables considered in the classification models. t-1, t and t+1 represent 3 consecutive positions of buoys over time.

**Variable**	**Formula**
Time interval (s)	time_t+1_—time_t_
Time interval before (s)	time_t_—time_t-1_
Time interval change (s)	time_t+1_—time_t-1_
Speed (m.s^-1^)	Distance_t,t+1_/time interval_t,t+1_
Speed before (m.s^-1^)	Distance_t-1,t_/time interval_t-1,t_
Acceleration (m.s^-2^)	2(velocity_t,t+1_-velocity_t-1,t_)/time interval_t,t+1_
Heading change (rad)	|heading|_t,t+1_-|heading|_t-1,t_
Min distance from a major port (km)	linear distance_t-Port_
Water temperature (°C)	temperature_t_
Water temperature before (°C)	temperature_t-1_
Water temperature change (°C)	temperature_t_- temperature_t-1_
Water temp. change / interval (°C.s^-1^)	(temp_t_- temp_t-1_)/time interval_t-1,t_

The methods tested were: a speed filter (VEL), multiple logistic regression (MLR), artificial neural networks (ANN) and random forests (RF). These methods range from fairly intuitive approaches (VEL, MLR; [23,24]) to sophisticated, ‘black-box’ models (ANN, RF) capable of representing complex interactions between variables without making assumptions regarding the distribution of the classification variables (ANN, RF;[25,26]), and of coping with noisy data and correlated classification variables (RF; [25,27]). In the case of the RF method, often described as robust to correlation among predictors [27], these may however induce a biased interpretation of the contribution of such variables to the model [28–30]. As our objective was not to build a good explanatory model but a good classifier of GPS buoy positions, we chose to include all available classification variables, regardless of their possible correlation. This is further discussed in S2 File.

### Configuration of classification models

The performance of the best model configuration for each of the 4 classification models was evaluated using cross-validation. The learning dataset was randomly split 100 times into a training dataset (used for model calibration) and a validation dataset (used to evaluate model performance) each containing 50% of the learning trajectories. During the calibration phase, each of the 100 training datasets was used to build an optimal version of the MLR, ANN, and RF models.

The full list of predictor variables can be found in Table 2. With the exception of the VEL model, which was manually calibrated based on the maximum “at sea” speed observed in the learning dataset, all model calibrations and predictions were carried out using R version R.2.14 [31] with the *caret* package (version 5.15–023, [32]) and its *train* function. The *train* function uses a bootstrap approach, with 200 iterations, to determine an optimal set of model configuration parameters (i.e., parameters that affect model structure and complexity, such as the number of hidden neurons in the ANN model; Table 3). For each of the 100 training datasets described above, 200 different random subsets are generated by resampling with replacement the training dataset, and then each given classification method is calibrated for each subset using all possible combinations of model-configuration parameter values. For each subset, the accuracy rate (fraction of correct predictions) and the Kappa statistic (which measures to what degree the prediction will be repeatable and reproducible) are computed using the remaining, unused part of the original training dataset. The set of configuration parameter values that maximizes the mean accuracy and mean Kappa among the 200 bootstraps is used to calibrate the given classification model to the entire training dataset. In the end, this procedure produces 100 optimized predictive classification models, one for each training dataset. The *train* function internally calls a different model for each classification method: the MLR and ANN method of the *nnet* package (version version 7.3–1, [33]), and the RF method of the *RandomForest* package (version 4.6–6, [34]).

**Table 3:** Classification methods used in to separate ‘at sea’ and ‘onboard’ positions of the buoys.

**Method**	**Features of interest**	**References**	**Parameters**
Multiple Logistic Regression (MLR)	intuitive, white-box	Dreiseitl and Ohno-Machado 2002	Weight decay w
Artificial Neural Network (ANN)	no assumption, complex non-linear relationships	Dreiseitl and Ohno-Machado 2002	Weight decay w
			Size s
		Joo et al. 2011	
Random Forest (RF)	no assumption, complex non-linear relationships, robustness to overfitting	Breiman et al. 2001	Randomly chosen variables mtry
		Cutler et al. 2007	

As the learning dataset is imbalanced in favour of “at sea” positions (86.2%), we also considered two approaches for correcting for imbalanced data: (1) using an optimal threshold other than 0.5 as the minimum probability required to declare a point “at sea” and (2) forcibly balancing the training dataset before model calibration. The first approach used maximization of sensitivity plus specificity [35] to determine a threshold for all classification methods other than VEL. The second approach was applied to the RF model as the RF algorithm used possesses an internal procedure to rebalance data. As neither of the two approaches improved overall model classification performance, they are not discussed further here, but details can be found in S1 file.

### Comparison of classification methods

The validation phase consisted of using the models calibrated on the 100 training datasets to predict the class of the positions in the corresponding 100 validation datasets. Classification model performance was evaluated through a balance of 5 indicators of performance based on minimization of the misclassification of “at sea” and “on board” positions (“position based” indicators, Table 4) and based on the ability to minimize the incorrect segmentation of trajectories (when sequences “at sea”—“on board” or “on board”- “at sea” occur along a trajectory) due to classification errors (“trajectory based” indicator, Table 4). 100 values of each indicator were calculated over the cross-validation procedure to obtain a distribution of their values. Pairwise comparisons of the performance of the models were then performed based on two sided t-tests (α = 0.05) using the speed filter (VEL) as the reference method. During this comparison phase, we made sure that each single position was correctly assigned a class “at sea” or “on board” through position based indicators such as the True and False Sea Rates (Table 4). However, as our objective was not only to correctly classify each single position but also sequences of “at sea” and “on board” positions, we ensured that improving position based indicator values was not inducing an over-segmentation problem. For this purpose, we put more emphasis on decreasing the segmentation rate than on increasing the TSR or decreasing the FSR as we considered less important to correctly classify a few isolated positions than to correctly capture a whole section of “at sea” or “on board” positions.

**Table 4:** Definition of position-based and trajectory-based indicators of performance for classification methods.

**Type**	**Indicator**	**Formula**	**Description**
Position based	Error rate	FB + FS / N_positions_	Accuracy of the classifier (no distinction of class)
	Precision	TS/ S_predicted_	Repeatability and predictive power
	True Sea Rate	TS / S_observed_	Sensitivity. Ability to detect S positions
	False Sea Rate	FS / B_observed_	1—Specificity. Ability to detect B positions
Trajectory based	Segmentation rate	$\frac{{\text{N}}_{\text{segments},\text{pred}}\;-{\text{N}}_{\text{segments},\text{obs}}}{{\text{N}}_{\text{segments},\text{obs}}}$	Inappropriate segmentation of the trajectories

S: at sea, B: on board, TB: True Boat; TS: True Sea; FB: False Boat, i.e., the number of positions incorrectly predicted to be on board; FS: False Sea, i.e., the number of positions incorrectly predicted to be at sea; N_segments_: number of segments over a GPS buoy trajectory; N_positions_: number of GPS buoy postions, obs: observed, pred: predicted

### Trajectory post-processing

The classification methods described above do not take into account the temporal relationship between successive buoy positions, but rather treat each position as independent of all others. This assumption can result in incorrect sequences of “at sea” and “on board” classes inconsistent with the fishing process. For instance, a sequence of the type ‘BSB’ is unrealistic as buoys are unlikely to be left at sea for only a few hours. Hence, post-processing of the outputs from the best classification model was performed to reclassify buoy positions in short “at sea” trajectories as being “on board” positions. During this procedure, we varied the maximum number of isolated, consecutive “at sea” positions to be reclassified as “on board” positions. For each maximum length for reclassification, we recalculated performance indicators (Table 4). Results with and without post-processing of predictions from the RF model were compared using a two-sided t-test of the indicators of performance (α = 0.05).

### Model application and data analysis

The best classification model including post-processing corrections was applied to the full buoy position dataset, and “at sea” and “on board” predictions were made for each position. Model predictions were then employed to detect potential fishing set positions assuming that transitions from “at sea” to “on board” potentially correspond to the retrieval of a dFAD and its buoy from the sea. Spatial patterns in fishing positions predicted by the model were compared to observed fishing positions as declared in fishing vessel logbooks. Note that predicted retrieval locations include some operations on floating objects that do not correspond to a fishing set (e.g., maintenance, buoy displacement to a different location or foreign buoy replacement), as well as buoys lost at sea due to the sinking of the attached floating object. 1-degree gridded density maps of observed and predicted fishing positions were created, and qualitative and quantitative comparisons between the two were carried out. These analyses were used both as a validation of the classification method and as a means to identify zones where endpoints of dFAD trajectories may not be related to fishing sets. Quantitative comparisons consisted of computing the Spearman correlation coefficient of observed and predicted densities in all grid cells containing at least one observed or predicted fishing set position.

Model results were then used to: (i) characterize dFAD trajectories (i.e. distance and time at sea), (ii) describe the spatial distribution of dFADs (using 1-degree gridded density maps in the Atlantic and Indian Oceans during 2007–2011), and (iii) calculate the fraction of time buoys spend outside historical fishing grounds, presumably representing ineffective fishing effort. In addition, possible dFAD beaching events were identified using the original, unclassified dataset by series of repeated geographical positions. The unclassified dataset was used to avoid any possible confusion in the classification algorithm between at port and beached positions. We assumed that at least 3 repetitions of the same position were necessary to identify a possible beaching event as occurrence of 2 repeated positions is known to be related to failures to correctly capture a GPS signal (see section *Buoys positions data and pre-processing*). Final results were obtained using 2 successive filters on these potential beaching-event positions. First, we eliminated positions located within 10 km of a port, assuming that these are likely to be simply fishing vessel anchorage points. Second, we eliminated positions located more than 5 km from land (accounting for 5.7% of all potential beaching events). These later “stopping points” may represent real shoaling events on offshore, shallow-water areas, but were considered more likely to be due to something other than shoaling, and, therefore, results were calculated with and without these points.

‘Ineffective’ dFAD fishing effort was described through 1-degree gridded density maps of buoys drifting outside historical dFAD fishing grounds, the proportion of fishing sets predicted outside fishing grounds, and the fraction of time a given dFAD spent drifting outside fishing grounds. Two definitions of historical fishing grounds were considered: the spatial distribution of catch under floating objects between 2006 and 2012 based on (1) the French fleet only and (2) all operating fleets. Fishing grounds of the corresponding fleet(s) were defined as all one degree grid cells containing at least one purse-seine fishing set.

## Results

### Classification model performance and selection

A speed threshold of 1.3 m.s^-1^ produces a classification of “at sea” positions with a True Sea Rate (TSR; see Table 4 for definitions of model performance statistics) of 99.3%. However, the False Sea Rate (FSR) of 43.3% indicates that almost half of the “on board” positions are classified as “at sea” (Table 5). Compared with the VEL model, True Sea Rate does not noticeably increase or decrease for any of the classification models tested in this study. For the MLR, ANN and RF (without and with post-processing of outputs) models, the error rate, the False Sea Rate and the segmentation rate all decrease while the precision increases. Among these indicators, the most important improvement is obtained for the False Sea Rate, which decreases to 24.2%, 17.8% and 12.3% in the MLR, ANN and RF (without post-processing) models, respectively. The segmentation rate for these 3 models decreases from a 143% increase in state transitions (i.e., predictions of ‘BS’ or ‘SB’ transitions relative to the true rate in the learning dataset) for VEL to +90.8, 93.2%, 62% for MLR, ANN, RF models respectively. Though the MLR, ANN and RF models produce similar values for True Sea Rate, all other indicators of performance indicate that the RF model performs considerably better than the MLR and ANN models, especially with regards to False Sea Rate, precision and segmentation rate (Table 5). Because of its superior performance, the RF model was chosen as the best classifier for dFAD trajectory data.

**Table 5:** Performance of the classification methods, as a mean of the indicator on the 100 cross-validation iterations for the VEL, MLR, ANN and RF method.

**Performance indicator**	**VEL**	**MLR**	**ANN**	**RF**
Error rate (%)	6.6	3.8 [-2.8;-2.7]	3.4 [-3.3;-3.1]	2.6 [-4.1,-3.9]
Precision (%)	93.4	96.2 [2.7;2.9]	97.1 [3.7;3.8]	98.0 [4.5;4.7]
True Sea Rate (%)	99.3	99.5 [0.1, 0.18]	99.0 [-0.4;-0.3]	99.0 [-0.4;-0.2]
False Sea Rate (%)	43.4	24.2 [-19.6;-18.7]	17.8 [-25.9,-25.1]	12.3 [-31.2,-30.6]
Segmentation rate (%)	142.9	90.8 [-54;-49.9]	93.2 [-52,5;-46.5]	59.3 [-86.6;-80.2]

For the MLR, ANN and RF methods, 95% confidence intervals of the difference between the indicator for method and the VEL method are given in square brackets.

**Fig 3:**
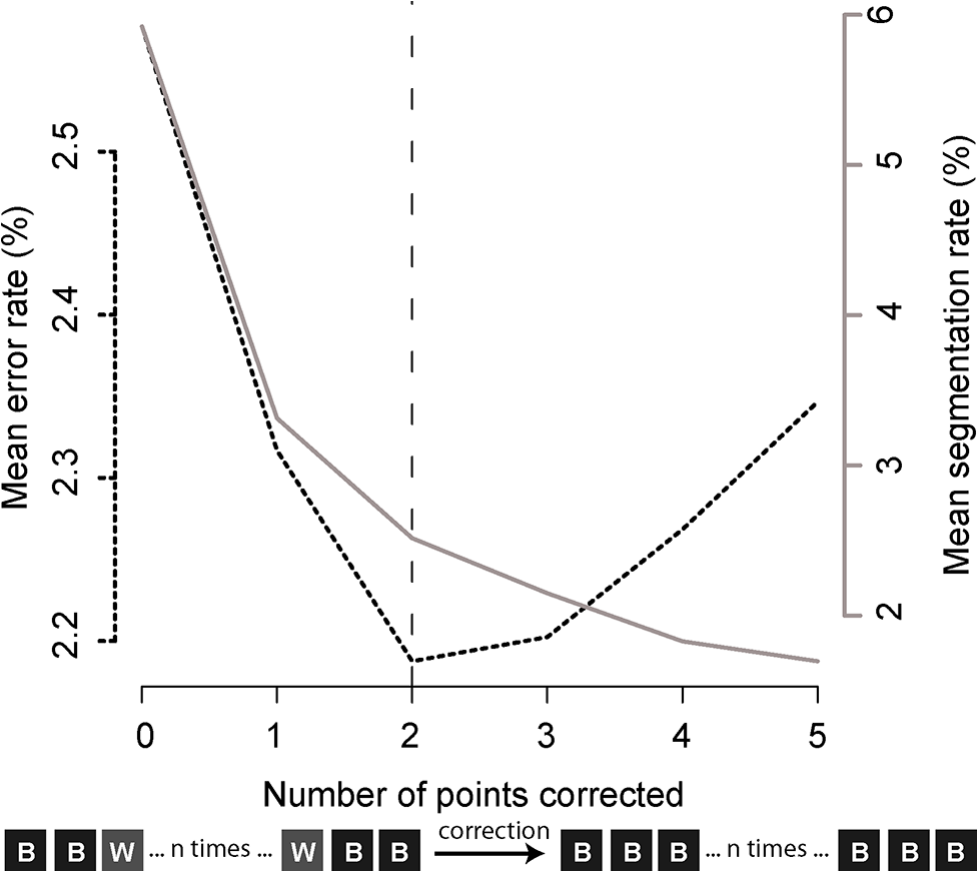
Mean error and segmentation rates over 100 cross-validation datasets for correcting between 1 and 5 isolated “at sea” positions.

Replacing RF predicted classification sequences of the type *BSSB* (i.e. two, isolated points classified as “at sea”) with *BBBB* considerably improves performance indicators. Error rate drops from 2.6% to 2.2% on average for the 100 validation datasets (Fig 3). This correction also significantly improves all other performance indicators (Table 5), notably reducing the segmentation rate from 60% to 25%. Using the RF model with post-processing correction, we predict that 15.5% of the full dFAD trajectory dataset consists of “on board” positions, showing the importance of separating “at sea” and “on board” positions before analysing patterns of dFAD use.

### Spatial patterns in dFADs

**Fig 4:**
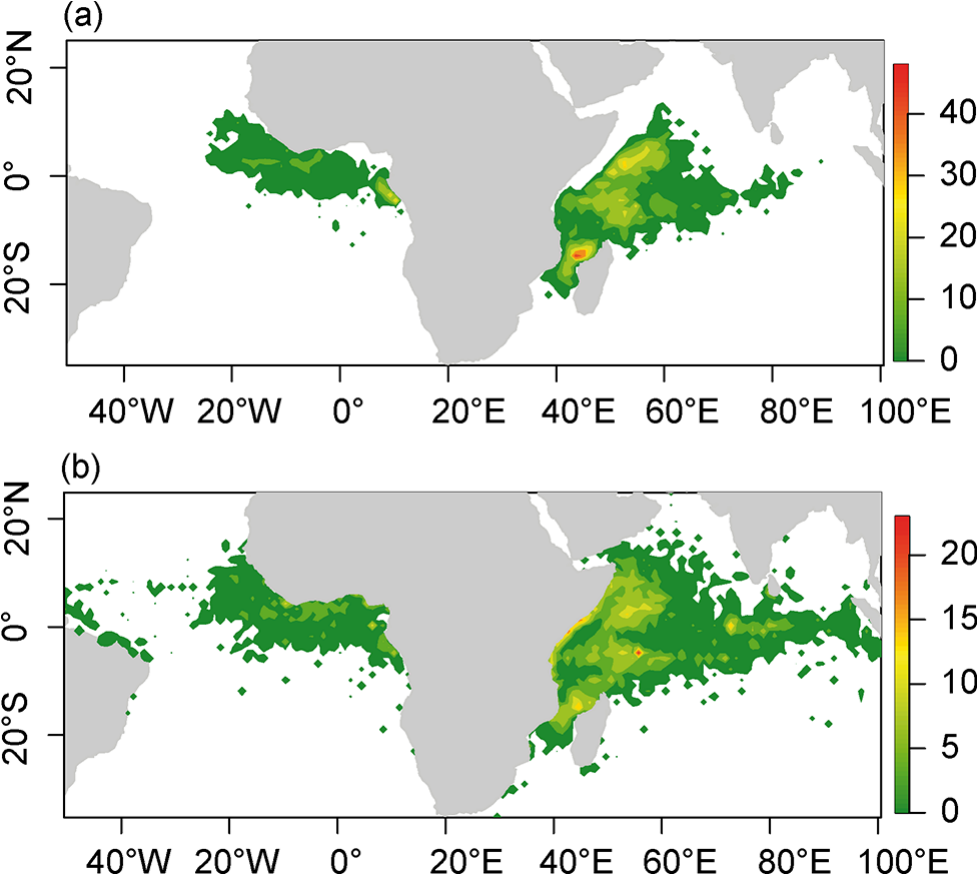
Smoothed mean densities of observed (as declared in logbooks, a) and predicted dFAD fishing sets (as derived from the corrected RF outputs, b) for the period 2007–2011. Densities were calculated on a 1° grid and smoothed using the two dimensional density estimation function *kde2d* of the MASS package in R (bandwidth chosen according to the rule-of-thumb provided in the function *bandwith*.*nrd*).

Overall patterns of potential dFAD fishing sets derived from the classified buoy data (i.e., ending points of “at sea” trajectories) are similar to the spatial pattern of fishing sets derived from vessel logbooks from French purse seiners over the period 2007–2011. The cross-correlation Spearman coefficient between observed and predicted spatial patterns of fishing sets is 0.64 (p < 0.001). More importantly, the main features and hotspots of the spatial distribution are correctly identified (Fig 4). dFAD-associated fishing sets, as declared by the skippers, are mainly concentrated from the Senegalese to the Gabonese coasts in the Atlantic Ocean, while they are mainly observed off Somalia and in the Mozambique Channel in the Indian Ocean. Predicted dFAD fishing grounds cover broader zones in the Indian and Atlantic Oceans than logbook data, extending into the western Atlantic and eastern part of the Indian Ocean where few fishing sets by French purse seiners occur. These differences may be attributable to deactivation of GPS buoys for dFADs that are drifting too far from fishing grounds, sinking of dFADs or dFAD use by fishing fleets for whom data is not available (e.g., artisanal fleets). However, zones of predicted fishing sets that are not observed in French purse seine logbook data generally have low densities of predicted dFAD trajectory endpoints, and principal fishing zones predicted from dFAD trajectories are largely consistent with logbook fishing sets.

### dFAD time and distance at sea

**Fig 5:**
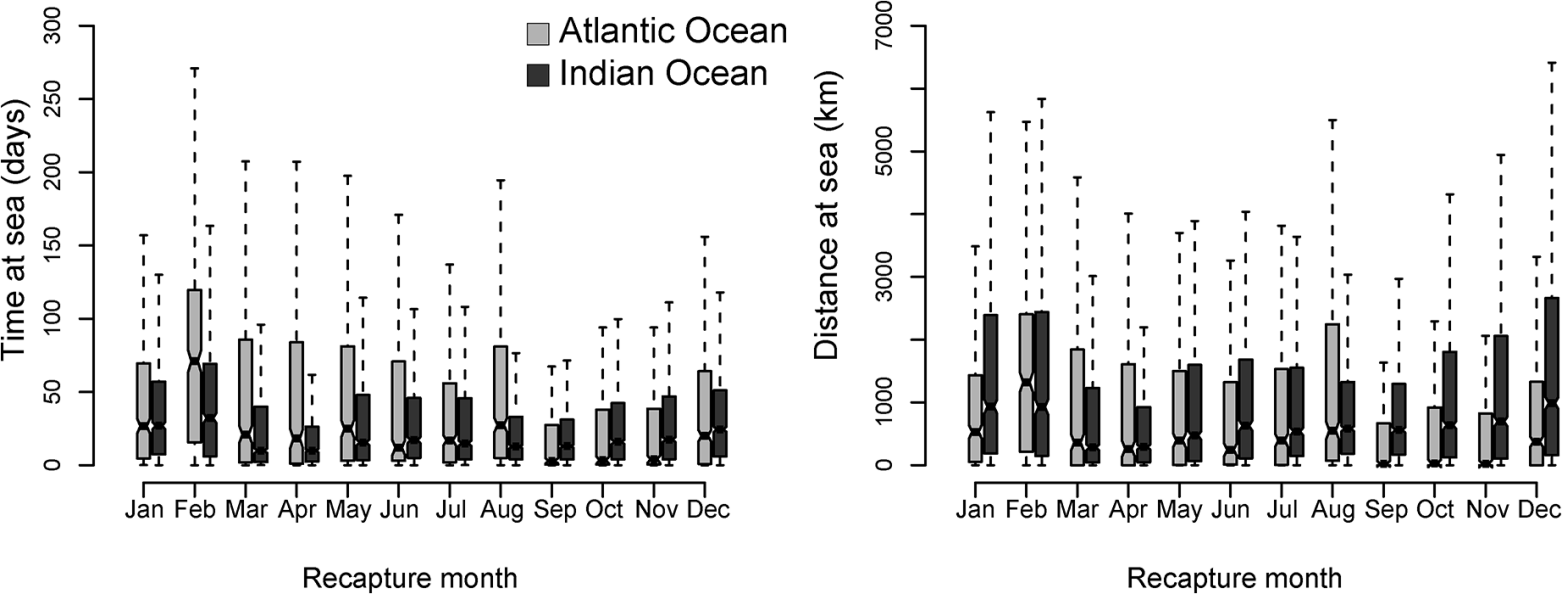
Time (a) and distance (b) at sea per ocean (in d and km) as a function of recapture month.

Predicted “at sea” portions of dFAD trajectories are on average 39.5 days long (standard deviation (SD) of 61.6, standard error (se) of 0.4), corresponding to a mean piecewise-linear drift distance of 1225.8 km (SD 1829.3, se 12.05), with both statistics showing important differences between oceans, years of release and months of recapture (Fig 5). In the Atlantic Ocean, both interannual and seasonal variability in time and distance-traveled at sea are important. Mean time at sea is 47.8 d (SD 69.6, se 0.89) with a minimum predicted time length of 1 hour and a maximum of 825 d (i.e. more than 2 years). Atlantic interannual variation in time at sea is important, e.g. with an average time at sea of 72.4 d in 2009 (SD 80.1, se 2.73) and 34.6 d in 2011 (SD 57.8, se 1.16). From February to November, days spent at sea decrease from 81 d on average (SD 82.9, se 3.6) to 29.9 d (SD 54.2, se 2.36). These monthly times at sea were significantly different (two-sided F test comparison of variances, α = 0.05: p-value<0.001). During the period September-November, distance at sea is the shortest of the year, with dFADs travelling 664.6 km (SD 1322.4, se 57.55) in November versus 1627.4 km (SD 1824.3, se 78.37) in February. Again, differences between months are significant (two-sided F test comparison of variances, α = 0.05: p-value<0.001). Note that the apparent high turnover rates of dFADs during the period September-November may also be related to frequent transfers of GPS buoys (when purse seiners replace a buoy found on a foreign dFAD with one of their own buoys).

In the Indian Ocean, time at sea (36.6 d, SD 58.2, se 0.44) is on average shorter than in the Atlantic Ocean, although the distance travelled at sea (1285.5 km, SD 1897.1, se 14.58) is higher. Variations also occur between years but with a lower magnitude than in the Atlantic Ocean, ranging from 32.6 d (SD 51.6, se 0.89) in 2011 to 45.7 d (SD 53.3, se 0.92) in 2008. dFADs retrieved in March-April and August-September generally spend less time at sea than those retrieved from December to February, with the shortest time at sea obtained for the month of April (28.4 d, SD 51.8, se 1.38) and the longest for the month of February (53.5 d, SD 73.0, se 2.43). These monthly times at sea were significantly different (two-sided F test comparison of variances, α = 0.05: p-value<0.001).

### Lost GPS buoys

**Fig 6:**
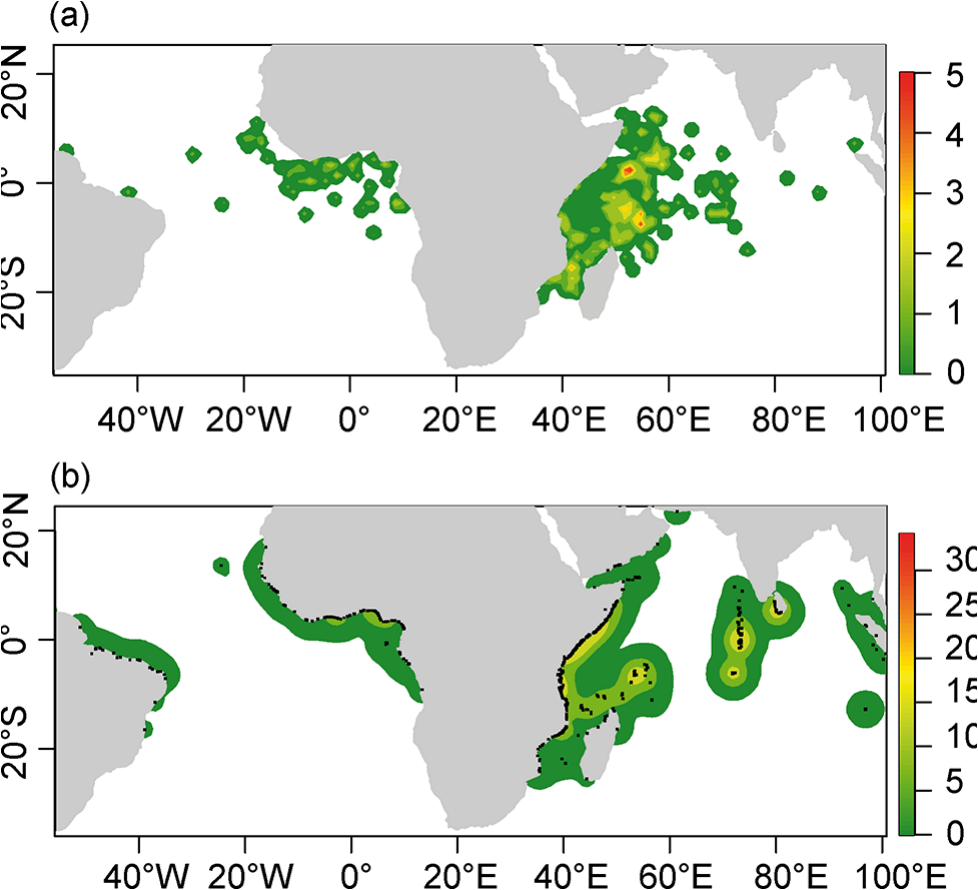
Smoothed densities of dFAD beaching events (b) and their corresponding deployments positions (a). Black dots correspond to individual beaching positions.

Putative beaching events, identified by positions that repeat at least three times far from a port, occur in 26.4% of the GPS buoy tracks, corresponding to 10.5% of the “at sea” trajectories in the dataset (a lower percentage because GPS buoys have more than one “at sea” trajectory). When distance to the coast is also taken into account, the percentage of beached at sea trajectories decreases to 9.9% (i.e., 5.7% of all putative beaching events occurred more than 5 km from the coast). More potentially beached GPS buoys are detected in the Indian Ocean (1328) than in the Atlantic Ocean (1128), in line with the larger number of dFADs deployed by the French fleet in this ocean. In the Atlantic Ocean, potentially beached buoys tend to concentrate in the Gulf of Guinea but some buoys also cross the entire ocean to strand on the Brazilian coast (Fig 6A). These dFADs have been deployed “at sea” off Abidjan, Tema, in the Gulf of Guinea and off Gabon (Fig 6A). In the Indian Ocean, beaching events occur over a wider set of zones, with Somalia, the Seychelles, the Maldives and Sri Lanka being the most important. Beaching events also occurred within the Marine Protected Area of the Chagos Archipelago (Fig 6B). Their deployment positions are mainly located around the Seychelles, in the Mozambique Channel and off Somalia (Fig 6B). As for the buoys found potentially stored at port (that are not part of the previous numbers), 7.3% are found far from a major landing port (Abidjan, Ivory Coast; Dakar, Senegal; Tema, Ghana; Victoria, Seychelles; Port Louis, Mauritius; Saint-Denis, Reunion Island; Diego Suarez, Madagascar; or Mombasa, Kenya), with this proportion being slightly higher in the Atlantic Ocean than in the Indian Ocean, consistent with the presence of more ports that are not used for tuna landings in the Atlantic Ocean. These “at port” buoys may correspond to buoys found by vessels that do not belong to the French purse seine fleet. Therefore, they could be considered as lost for the French fleet, as purse seiners rarely have the possibility to retrieve buoys from such minor ports (when purse seiners from other major, industrial fleets find and replace French buoys with one of their own buoys, the French buoy is generally returned to the docks of one of the major ports).

### ‘Ineffective’ dFAD effort

**Fig 7:**
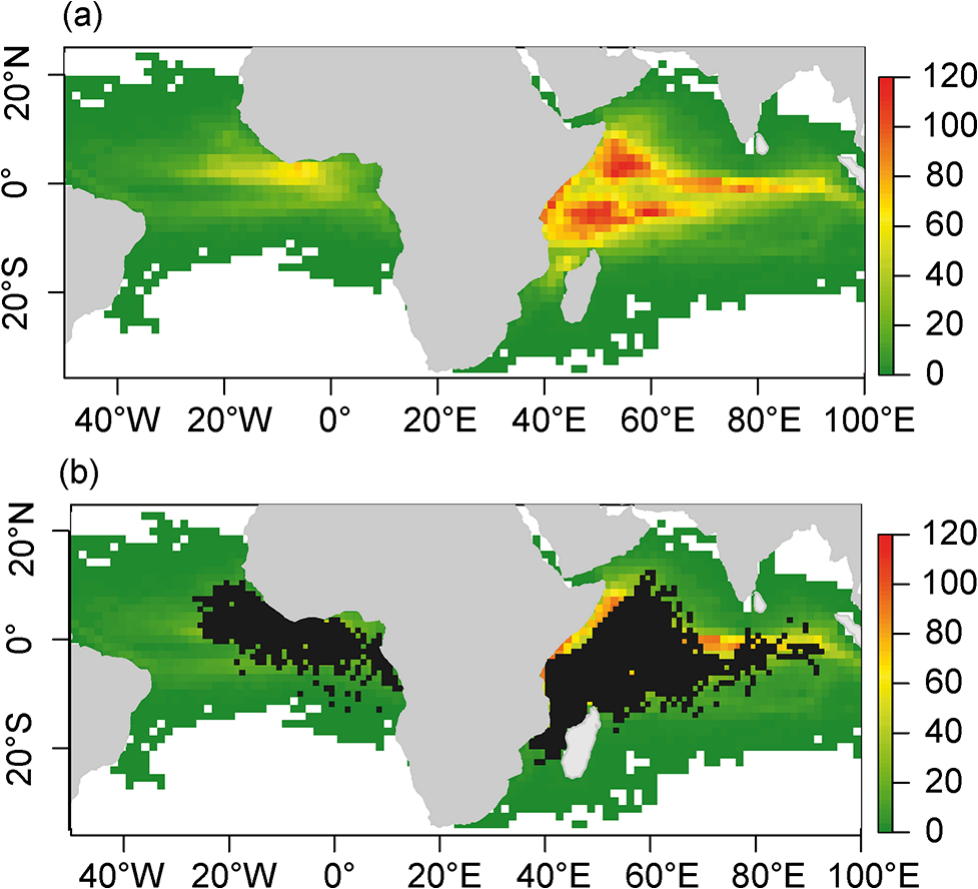
Mean yearly dFAD density (a) and ineffective dFAD effort (b) for the period 2007–2011. Black areas correspond to 1° grid cells where at least one French or Spanish fishing set occurred over the period 2006–2012.

A total of 6,563 GPS buoys (i.e. 68.4% of the dataset) were found to be drifting outside French fishing grounds (see Fig 4A for the location of French fishing sets on dFADs) at least once during their whole “at sea” set of trajectories. By comparison, with fishing grounds based on all fleets for the period 2006–2012 (Fig 7B), this number decreases to 5,420 (57% of the dataset). Though the average fraction of total drift time spent outside fishing grounds is relatively small (3.05% for French fishing grounds; 2.2% for all fishing grounds), for some buoys, the time spent outside fishing grounds is extensive. For example, 20.6% of the drifting trajectories spent less than 50% of the time inside French fishing grounds (8.5% if all fishing grounds are considered). Main zones of dFADs travelling outside French fishing grounds are the area around the port of Tema (Ghana) and a large area east of the fishing ground in the Atlantic Ocean, as well as the Maldives, the eastern coast of Sumatra and the area adjacent to the coast of Somalia in the Indian Ocean (Fig 7).

## Discussion

Our analyses of the spatio-temporal distribution of dFAD trajectories both complement existing data on tuna fishing activities, as well as provide new, previously-unavailable insights into purse seine strategy and potential impacts of fishing. In particular, we provide for the first time information on the principal characteristics of dFAD use (i.e. density, turn-over, travelled distance, time at sea, and variability in time and space), essential for improving the monitoring and management of fishing effort exerted by purse seine fleets in the Atlantic and Indian Oceans. Though our dFAD buoy positions are characterized by irregular time-steps, occasional abherant data and mixing of “at sea” and “on board” states, the classification methodology described here is able to reconstruct “at sea” trajectories with a relatively high level of accuracy. The best classification methodology, consisting of a random forest binary-classification model followed by post-processing to remove short “at sea” trajectories consisting of just one or two “at sea” positions, has an error rate of just 2.2%. Nevertheless, 25% more “at sea” trajectories are predicted than are observed, suggesting that improvements to reduce the trajectory segmentation rate are still possible (see end of Discussion).

Seasonal variation in dFAD mean times at sea are consistent with known patterns of purse seine fishing activity [16,17,20], though variability in trajectory time length is very high among “at sea” trajectories. In both oceans, during periods when purse seiners concentrate on dFAD-fishing, times at sea are shorter than during seasons when fishers mainly target free-swimming tuna schools, suggesting higher rates of dFAD deployments and buoy transfers during these periods. Times at sea are shorter in the Indian Ocean than in the Atlantic, but the reverse is true for distance travelled by dFADs. These results are potentially explained by the stronger ocean currents and ocean variability in the Indian Ocean (e.g., in areas off Somalia), than in Atlantic fishing grounds [36,37]. They may also be explained by differences in the design of dFADs between oceans, with the length of the net hanging down beneath the bamboo raft reaching up to 70-100m in the Atlantic Ocean compared to only 30-50m in the Indian Ocean [38]. The former is considered to reduce distance travelled for Atlantic Ocean dFADs due to increased drag from slow-moving water masses below the thermocline [9]. Finally, differences in time at sea may be related to differences in concentration of purse seiners on fishing grounds that reduce the probability of a raft to be stolen and its buoy to be transferred, thereby increasing “apparent” time at sea in the Atlantic Ocean. High variability among “at sea” trajectories is consistent with the unpredictable nature of dFAD use: dFADs may be rapidly stolen by other vessels, drift for longer or shorter periods before aggregating tuna, or drift outside fishing zones but continue to be monitored for months by skippers.

These initial results on time and distance travelled at sea form a foundation that could be used to model dFAD trajectories, understand the mechanisms underlying these spatio-temporal differences, and hopefully develop management strategies to limit negative impacts on pelagic ecosystems. For example, if the time a given dFAD spends at sea results in changes in catch, bycatch levels or higher probabibilities of ghost fishing (see below), restrictions such as a minimal or a maximal time at sea could potentially be implemented. Distance at sea is also crucial to test the efficacy of spatialized management tools. For example, if dFADs travel a long distance from their deployment position, and tuna remain “trapped” in the array of moving dFADs, they may be extracted from closed areas to be fished elsewhere. These results may also be useful for assessing potential for dFADs to act as ecological traps for tuna, disturbing normal tuna behaviors and leading to reduced survival or growth [13,14].

With the objective of identifying “ineffective” dFAD fishing effort, we measured the proportion of time “at sea” trajectories occuring outside established fishing grounds. A large proportion of dFADs travel outside fishing grounds during part or all of the time spent drifting at sea, with only 32.3% spending 100% of the time inside French fishing grounds (45.2% if all fleets are considered). Of the dFAD trajectories that are found to be always travelling outside French fishing grounds, 27.9% are inside fishing grounds based on all fleets, and, therefore, this fishing effort may eventually be exploited by non-French industrial fishing fleets. In some cases, such as dFADs passing through the Somali EEZ, these dFADs may be recovered at a later date elsewhere. In others, such as dFADs west of 30°W or east of 80°E, these floating objects are unlikely to be recovered by purse seiners. In such cases, they may represent ineffective or lost fishing effort, or they may eventually be used by other tuna fisheries (e.g., artisanal fisheries of coastal states) in the region. It is unknown what impact these drifting objects may have on the pelagic environment, but some authors have hypothesized that they may represent an ecological trap for tuna and other pelagic species, affecting fish condition, growth and mortality, and moving fish schools outside of prime habitat areas [13,14,39]. In addition, active or abandoned dFADs could result in high ghost fishing mortality of turtles and sharks through entanglement in the netting that hangs underneath the rafts [40–43]. Modifications in the design of dFADs to reduce risks of entanglement without decreasing their capacity to aggregate tunas have been proposed and recently implemented for the European purse seine fleet [38,44]. Defining purse seine dFAD-fishing effort as directly proportional to the density of dFADs is of course simplistic, but provides a useful alternative to conventional measures of fishing effort, such as vessel search time or number of fishing sets, which are not capable of estimating fishing impacts that occur in the absence of fishers.

Another important question regarding the use of dFADs is what is the eventual fate of lost buoys, and in particular, what impact beaching events may have on coastal environments via their contribution to coastal marine debris. Given that dFADs generally include a significant subsurface structure, including filaments up to 70m in length [38], this contribution may be non-negligible. Our analyses indicate that a non-negligible fraction (9.9%) of dFAD deployments (inferred from “at sea” trajectories) do eventually end up beached. Given estimates of about 15–20,000 total [45] dFADs annually deployed in the two oceans, this would suggest around 1,500–2,000 beaching events per year, with significant portions of these beaching events occurring in potentially sensitive habitat areas, such as the coral reefs of the Maldives Seychelles, or the Chagos [46]. This number could be even higher, as we consider here only dFADs close to coastlines, whereas dFADs may also be retained on offshore shallow areas (though these are relatively rare in the Atlantic and Indian Oceans). Mitigating for these impacts by avoiding deployment zones and time periods with a high probability of leading to a beaching event may be possible. However, in the Indian Ocean, for example, this would greatly impact fishing activities during one of the most important seasons for the tuna fishery, as beached dFADs are mainly those that are used to prepare for dFAD fishing off Somalia. In this area, the absence of bilateral agreements allowing fishing in Somalia EEZ, the presence of piracy, the strength of the currents and the intensity of dFAD fishing may explain the high number dFADs lost onshore. This example serves as an illustration of how classified dFAD trajectory data can be used to assess dFAD impacts on fragile marine ecosystems and derive appropriate spatialized management tools based on dFAD deployment zones. Though preliminary, the results obtained could contribute to building a goal-based and transparent criterion for the regulation of dFAD use in time and space.

These results on dFAD spatio-temporal patterns and impacts are all derived from our classification methodology. This methodology is supported by a comparison of four methods to correctly identify “at sea” or “on board” states of dFAD buoys. Ideally, the correct prediction of the class of a given GPS buoy position would have relied on a simple, transparent decision rule. For instance, as purse seiners travel most of the time faster than ocean currents, a dFAD position could be classified as “on board” using an appropriate speed threshold. Though such a speed filter is among the most efficient methods to identify true “at sea” positions (TSR), the false “at sea” detection rate (FSR) for this method is considerable: 43.3%. This high error rate undoubtedly results from periods when the fishing vessel speed is low, for example during fishing sets and potentially at night. Due to this lack of a clear separation between vessel and dFAD drift speeds, more complex decision rules are necessary to classify dFAD positions.

By comparison, the Random Forest (RF) method produces the lowest mean error rate (2.6% versus 6.6% for VEL), lowest False Sea Rate (12.3% versus 43.3% for VEL) and lowest segmentation rate (59.3% versus 142.9% for VEL) of all methods considered, and maximizes the precision while maintaining a very high True Sea Rate. Though drift speed was consistently the strongest predictor of buoy state, other variables, such as acceleration, heading change, water temperature and distance to port, also contributed to the classification algorithm (Fig A in S2 File). Furthermore, the contribution of these variables to the classification algorithm is often non-linear (Fig C in S2 File). This explains the significant improvement in performance statistics for the more-sophisticated, non-linear algorithms integrating a full suite of predictor variables, such as ANN and RF. Multiple logistic regression (MLR) performances could have been improved by considering higher-order and interaction terms. Adding such terms would undoubtedly improve performance measures for this method, but there is little a priori basis for choosing the number and maximum order of such terms. Flexible, non-linear classification methods, such as RF and ANN, provide a clear advantage in this sense.

Because of the properties of the four methods tested in this study, the higher performances of the RF could have been anticipated. However, our aim was not only to identify the best classification method, but also to assess trade-offs in terms of model transparency and computational time. In this context, RF produces a non-negligible improvement in performance indicators that justifies its use, though this comes at a computational cost (~3–4 hours computational time to classify all currently-available dFAD position data with RF, versus ~10 minutes for MLR).

Overall, the False Sea Rate indicates that the RF model is highly efficient at identifying when the buoy is drifting at sea. Nevertheless, erroneous splitting of “at sea” or “on board” trajectory segments as a result of misclassifications remains important. For example, the RF model predicts 59.3% more trajectory pieces than observed in the training dataset. Though post-processing to remove very short “at sea” trajectory segments reduces the segmentation rate from 59.3% to 25.2% and improves several other performance indicators, over-segmentation remains non-negligible. Analyses of dFAD trajectories based on considering sequences of “at sea” or “on board”, such as mean time at sea, drift displacement distances or “at sea” trajectory start and end points, are probably biased in our results. This likely partially explains model predictions of very short “at sea” trajectories (e.g., <1 d), as well as putative fishing sets outside of purse seine fishing grounds. Though the correlation between observed and predicted fishing maps is high and important hotspots for dFAD fishing are identified by the RF corrected model, methodological improvements to reduce these biases are an important area for future developments.

There are a number of methodological approaches that may improve our analyses of dFAD spatio-temporal use patterns. One possibility is to use a learning dataset that is balanced in terms of number of “at sea” versus “on board” positions. This approach was tested when developing our classification model, but did not improve results (see S1 File). A balanced learning dataset is generally desirable in cases where either one prefers to err in favour of the minority class (e.g., when prediction the species distribution of a rare, endangered species) or one believes that the true prevalence of the minority class is higher than what is observed in the learning dataset [47,48]. Neither of these is the case for our dataset. Furthermore, balancing the learning dataset does not contribute to taking into account the temporal relationship between successive observations (see following paragraph).

Performance indicator improvements due to post-processing corrections to the RF model outputs suggest that the sequence of “on board” and “at sea” states in buoy trajectories is informative. Classification methods used here take into account the temporal relationship between position measurements only partially, via several variables (e.g., speed, acceleration, heading change, etc.) that are computed using information at previous and succeeding time steps. If the temporal correlation between the successive positions of a GPS buoy could be measured, integration of these correlations in the classification model may eliminate many extremely short dFAD “at sea” trajectories because such short deployments would be unlikely. Applying standard methods that integrate this type of information for classification purposes, such as Hidden Markov and Hidden Semi-Markov Models (HMM and HSMM), could be an alternative to the RF post-processing solution adopted in this study [49]. Similar to the discriminative methods examined here, these approaches model the relationship between the probability of being “on board” or “at sea” based on observations, such as the speed and the acceleration at given time. In addition, they consider that the probability of being in a given state at a given time depends on the past states. HSMM, in particular, considers the probability of being in a given state as a function of the time already spent in this state [49]. The use of HSMMs is not trivial in our case due to the highly irregular timesteps of dFAD trajectories and the high computational costs involved in applying these methods to large datasets. Furthermore, when fishing vessels concentrate in the same area, the probability of a dFAD to be found and its buoy to be transferred after a short drift is higher. Short “at sea” sections of trajectory during periods of intense dFAD fishing may, therefore, be real events and applications of HSMM to these data must take this seasonality into account.

The simplest and most direct solution to these issues would be to increase availability to information on deployment and recovery events of individual dFADs. Though classification schemes like the ones presented here are likely to remain valuable as checks of reported information and as corrections for missing data (e.g., GPS buoy transfers between different national fleets) or data limitations, analytical power would be significantly increased by direct access to data on these dFAD-related fishing activities. Information on dFAD transfers, visits, etc. has been recently added to logbooks of French purse seiners (since January 2013), and therefore it may soon be possible to use these data in combination with the classification and analysis approaches presented here to develop a suite of indicators of spatio-temporal intensity of dFAD use. In this context, the analyses of dFAD use presented here represent a necessary first step to designing effective management strategies for dFAD fishing.

## Supporting Information

S1 File Development of the classification models (DOCX)

S2 File Details on the RF outputs (DOCX)
