# Supplementary material for: Large-Scale Examination of Spatio-Temporal Patterns of Drifting Fish Aggregating Devices (dFADs) from Tropical Tuna Fisheries of the Indian and Atlantic Oceans
Source: PLoS One. 2015 May 26;10(5):e0128023. doi: 10.1371/journal.pone.0128023 (PMC4444087; doi:10.1371/journal.pone.0128023)
Supplement: S1 File — (DOCX) [file pone.0128023.s001.docx]

**Supplementary Information S1: Development of the classification models**

Our dFAD learning dataset is heavily imbalanced in favour of “at sea” positions (86.2% of the 19,927 positions). It is at time advantageous to take into account the structure of the learning dataset when building a classification model [1]. In our case, this could prevent the classification models from being driven by the “at sea” class and could reduce the prediction errors for the “on board” class. We tested two methods for dealing with an imbalanced training dataset: (1) the use of an optimal threshold probability other than 0.5 for classifying a position as “at sea”, and (2) a “balanced Random Forest” method.

*Optimal threshold via maximization of sensitivity+specificity*

Separating “on board” and “at sea” GPS buoy positions is a classical binary classification problem. In such problems, it is at times necessary to use a detection threshold of the binary classifier other than the default of 0.5 (meaning that positions with a probability of being at sea greater than 50% are considered at sea). We examined the impact of optimising the detection threshold by maximization of model sensitivity (its ability to detect true positives, in our case the positions “at sea” that are correctly classified as “at sea”, referred to in the text as the “True Sea Rate”) plus specificity (its ability to avoid false positives, in our case the positions “on board” that are incorrectly classified as “at sea”, referred to in the text as the “False Sea Rate”, [2]. Optimal thresholds were determined using the *optimal.thresholds* function of the R package *PresenceAbsence*. We present here the results obtained for the RF model with max sensitivity + specificity,, but maximization of the detection threshold for the two other classification models (MLR and ANN) and/or using a different optimal threshold selection approach (for example, maximization of Kappa) produced similarly small changes in model performance statistics.

The procedure was applied over the 100 cross-validation iterations. 100 values of the optimal threshold were obtained and used to calculate the corresponding 100 values of each indicator of performance (True Sea Rate, False Sea Rate, error rate, precision and segmentation rate).

**Table A.** Outputs of the RF model, with or without optimal threshold analysis

| **Performance indicator** | **RF without correction**  **(threshold=0.5)** | **RF with optimal threshold**  **(mean threshold=0.46)** |
| --- | --- | --- |
| Error rate | 2.6 | 2.5 [0.07; 0.2] |
| Precision | 98.0 | 97.9 [-0.06;0.2] |
| True Sea Rate | 99 | 99.2 [-0.27;-0.09] |
| False Sea Rate | 12.3 | 12.9 [-0.98; 0.1] |
| Segmentation | 59.3 | 55.1 [1.5; 6.8] |

Using an optimal threshold did not considerably change performance indicates. Although the other indicators of performance were improved, mean False Sea Rate of the RF was increased from 12.3 to 12.9. Given the limited value of threshold optimisation for the performance indicators we are interested in, we chose to use the default 0.5 threshold in the main text of the manuscript.

*Balanced Random Forest*

Using the “sampsize” argument in the randomForest function in R, the relative ratio between “at sea” and “on board” data in the training dataset was fixed at values varying from 9:1 (close to what is observed on average in our training dataset) to 1:1, corresponding to a dataset balanced between the “at sea” and “on board” positions.. The 5 indicators of performance were computed for each ratio based on 100 cross-validation iterations. This allowed us to test the effect of a wide range of structures of the learning dataset on the two possible types of misclassifications.


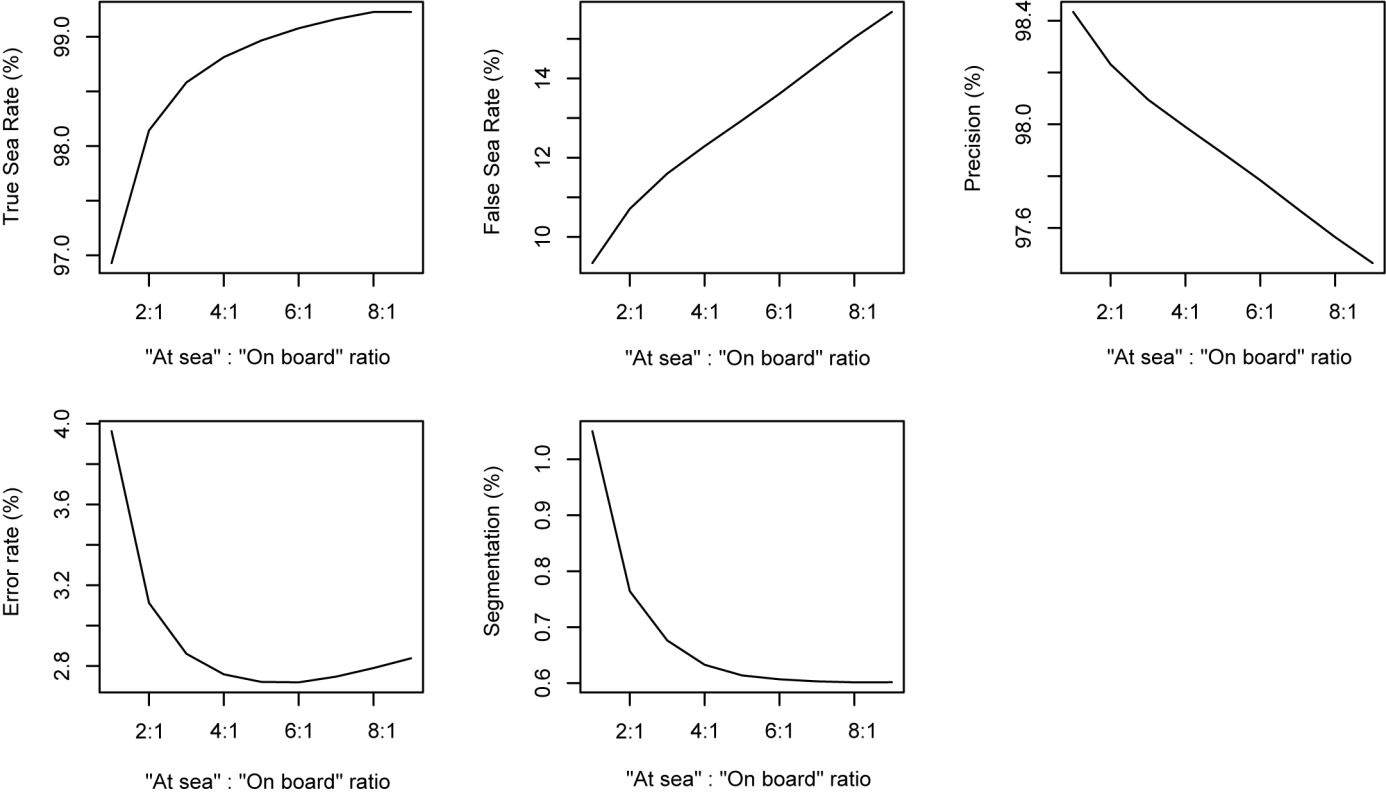


**Figure A.** Performance indicators according to changes in the ratio of “at sea” and “on board” positions in the training dataset.

Not surprisingly, balancing the training dataset introduces trade-offs between the different performance statistics. The False Sea Rate (FSR) decreases from 15.7% to 9.3% as the ratio “at sea”:”on board” is reduced from 9:1 to 1:1 (Fig. A). This means that the confusion of “on board” positions with “at sea” positions decreases when the balance of the two classes increases in the dataset. Similarly, balancing the training dataset results in increased precision. However, using a more balanced dataset results in reductions of the True Sea Rate and the overall error rate. Furthermore, reducing the ratio “at sea”:”on board” from 9:1 to 1:1 increased the segmentation rate from 60% to over 100% (Fig. A).

These results can be used to choose the desired relative costs of each type of misclassification. If our objective had been only to equally correctly classify “at sea” and “on board” positions, a good strategy would have been to choose a ratio “at sea”: “on board” of 2:1 as it greatly improves the False Sea Rate without decreasing too much the True Sea Rate. Another option would be to use a ratio of 5:1, that produces similar Error and Segmentation Rates and slightly reduces the False Sea Rate without decreasing too much the True Sea Rate and the precision. However, our objective was not only to correctly classify each independent position but also to ensure that the sequences of “on board” and “at sea” positions is correct along a given GPS buoy trajectory. For the present study, correctly classifying a few isolated positions is far less important than correctly classifying a whole “at sea” or “on board” section of trajectory. Besides, it is important to keep in mind that the classified trajectories are only useful for their “at sea” sections, as we already have access to vessel trajectories through Vessel Monitoring System (VMS) and “on board” sections only cover a fraction of fishing vessel tracks. As a consequence, overall, balancing the dataset only marginally improves the performance indicators most important for this dataset: True Sea Rate and the segmentation rate. Balancing the training dataset improves the detection of rare events such as “on board” positions , but reduces the ability of the method to detect the more common “at sea” positions. As we are most interested in properly detecting “at sea” positions and maintaining a low segmentation rate, we chose not to balance the dataset in analyses presented in the main text of the manuscript.

1. Meynard CN, Kaplan DM. The effect of a gradual response to the environment on species distribution modeling performance. Ecography. 2012;35: 499–509. doi:10.1111/j.1600-0587.2011.07157.x

2. Jiménez-Valverde A. Insights into the area under the receiver operating characteristic curve (AUC) as a discrimination measure in species distribution modelling. Glob Ecol Biogeogr. 2012;21: 498–507. doi:10.1111/j.1466-8238.2011.00683.x
