# Supplementary material for: Large-Scale Examination of Spatio-Temporal Patterns of Drifting Fish Aggregating Devices (dFADs) from Tropical Tuna Fisheries of the Indian and Atlantic Oceans
Source: PLoS One. 2015 May 26;10(5):e0128023. doi: 10.1371/journal.pone.0128023 (PMC4444087; doi:10.1371/journal.pone.0128023)
Supplement: S2 File — (DOCX) [file pone.0128023.s002.docx]

**Supplementary Information S2: Details on the RF outputs**

*Calibration of the RF model*

**Table A**

Results of the bootstrap calibration procedure. The optimal value of the parameters has been chosen based on a maximization of the accuracy (minimization of the error rate) obtained for a minimal value of Kappa.

| Method | Accuracy  (1–%error) | Precision  (Kappa) | Tested parameter values | Calibrated parameters |
| --- | --- | --- | --- | --- |
| MLR | 0.963 | 0.818 | decay= 0, 1e-6, 1e-5, 1e-4, 1e-3 | decay=1e-4 |
| ANN | 0.968 | 0.848 | decay= 0, 1e-6, 1e-4, 1e-2, 1e-1, 1  size= 1, 2, …, 10 | decay=1e-4, size=9 |
| RF | 0.978 | 0.899 | mtry= 1, 2, …, 5 | ntrees=1500, mtry=4 |

*Interpreting the RF model outputs*

Though the focus of the present study is to build a good classifier of “at sea” and “on board” GPS buoy positions, it might be of interest to better understand the structure of the RF model. For example, understanding the contribution of each predictor variable could explain the performances of the RF model, as compared to a simple VEL method. The RF method provides a measure of the relative importance of predictor variables included in the model. The mean decrease in Gini Index tends to indicate that important variables are the speed at the previous time step as well as the speed at time t (Figure A). However, this metric may not be the best in our case. Figure B indicates that some of the predictor variables such as speed variables or temperature variables are correlated or highly correlated (Kendall’s tau coefficient, used for its non-parametric nature, of 0.63 and 0.81 respectively). In such a case, the use of conditional Random Forest and the corresponding mean decrease in accuracy would be more indicated [1,2]. Indeed, when predictor variables are correlated, mean decrease in accuracy is biased and more weight is given to correlated variables [3]. Strobl et al. proposed an alternative method for assessing predictor variables importance in the case of correlation. However, as we are more interested in building a good classifier than in interpreting the RF outputs, this may not be a major concern for this study.


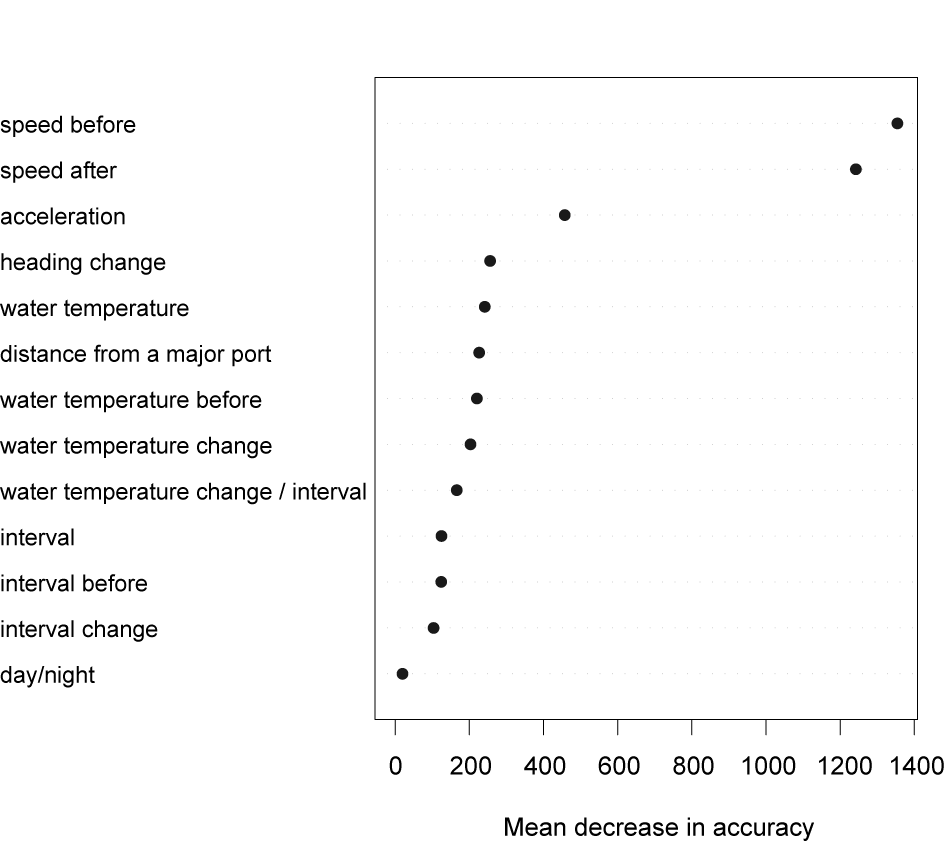


**Figure A:** RF model variable importance


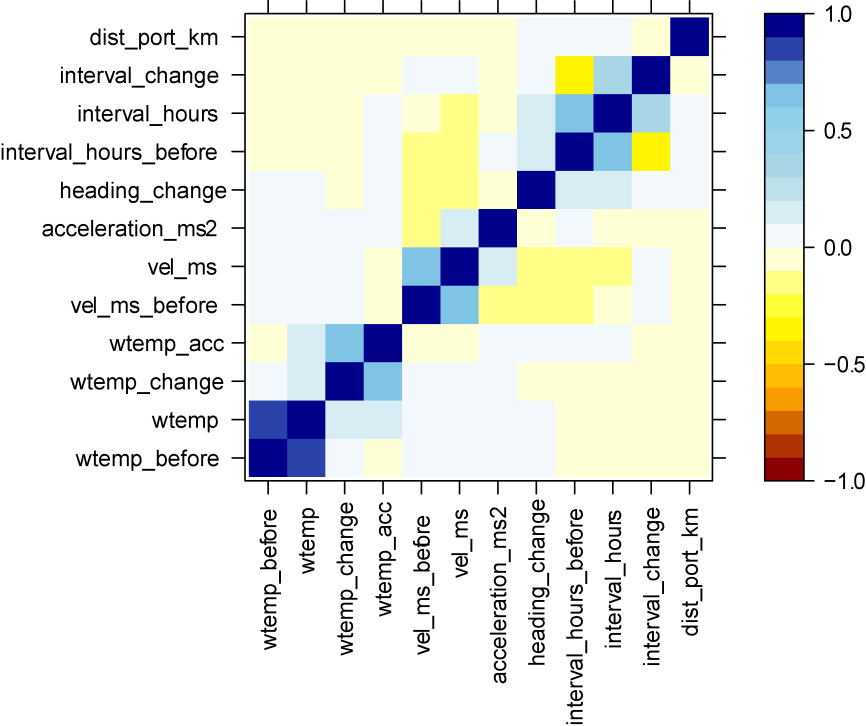


**Figure B:** correlated predictor variables included in the RF model (Kendall’s tau coefficient)


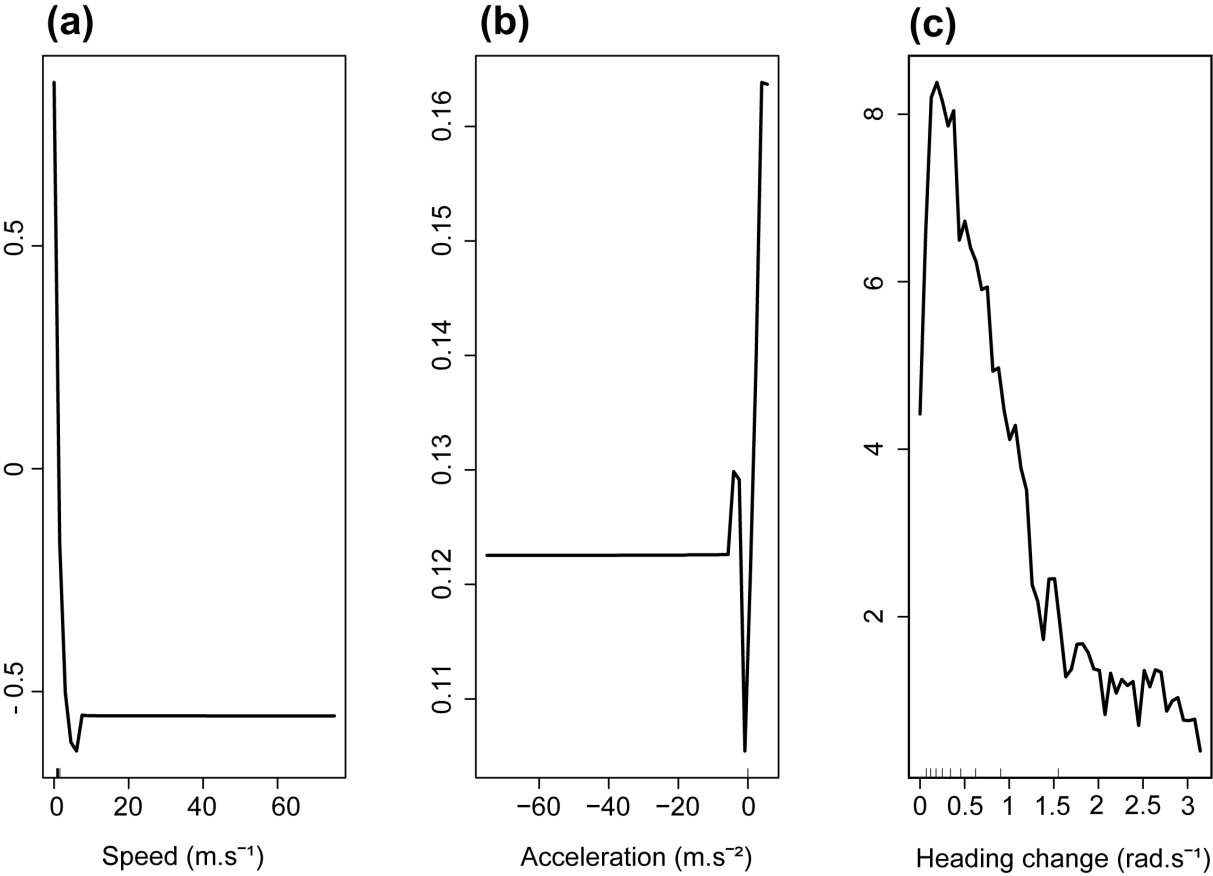


**Figure C:** Examples of partial dependence plots for important classification variables. These plots can assist in the detection of the values used to build the decision rule in the RF model.

*Correction of the RF model*

**Supplementary table B**

Results of the RF outputs postprocessing (complement of Figure C)

| Performance indicator | BSB | BSSB | BSSSB | BSSSSB | BSSSSSB |
| --- | --- | --- | --- | --- | --- |
| Error rate (%) | 2.3 | 2.2 | 2.2 | 2.3 | 2.4 |
| Precision (%) | 98.4 | 98.5 | 98.6 | 98.7 | 98.7 |
| True Sea Rate (%) | 99.0 | 99.0 | 98.8 | 98.7 | 98.6 |
| False Sea Rate (%) | 10.2 | 9.0 | 8.6 | 8.2 | 8.2 |
| Segmentation rate | 33.1 | 25.2 | 21.5 | 18.3 | 17.0 |

1. Strobl C, Hothorn T, Zeileis A (2009) Party on! A New, Conditional Variable Importance Measure for Random Forests Available in the party Package. R J 1: 14–17.

2. Strobl C, Boulesteix A-L, Kneib T, Augustin T, Zeileis A (2008) Conditional Variable Importance for Random Forests. BMC Bioinformatics 9: 1471–2105.

3. Nicodemus KK, Malley JD, Strobl C, Ziegler A (2010) The behaviour of random forest permutation-based variable importance measures under predictor correlation. BMC Bioinformatics 11: 110. doi:10.1186/1471-2105-11-110.

4. Cutler DR, Edwards TC, Beard KH, Cutler A, Hess KT, et al. (2007) Random Forest for Classification in Ecology. Ecology 88: 2783–2792. doi:10.1890/07-0539.1.
